# Advances in the Separation and Detection of Secondary Organic Aerosol Produced by Decamethylcyclopentasiloxane (D_5_) in Laboratory-Generated and Ambient Aerosol

**DOI:** 10.1021/acsestair.3c00073

**Published:** 2024-03-27

**Authors:** Jeewani N. Meepage, Josie K. Welker, Claire M. Meyer, Saeideh Mohammadi, Charles O. Stanier, Elizabeth A. Stone

**Affiliations:** † Department of Chemistry, University of Iowa, Iowa City, Iowa 52242, United States; ‡ Department of Chemical and Biochemical Engineering, University of Iowa, Iowa City, Iowa 52242, United States

**Keywords:** molecular tracers, volatile methyl siloxane, secondary aerosol, fine particulate matter, oxidative flow reactor, SOA

## Abstract

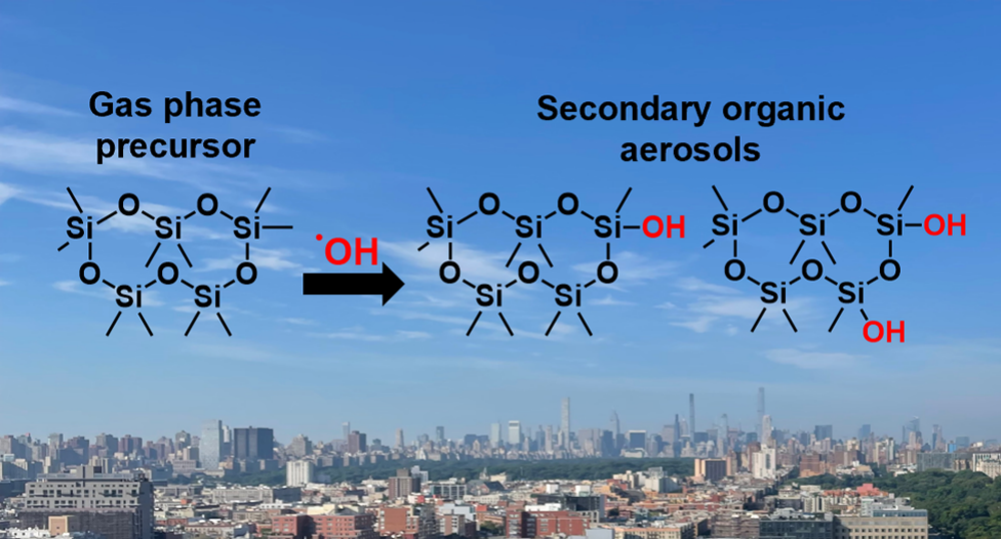

Decamethylcyclopentasiloxane (D_5_), a common ingredient in many personal care products (PCPs), undergoes oxidation in the atmosphere, leading to the formation of secondary organic aerosol (SOA). Yet, the specific contributions of D_5_-derived SOA on ambient fine particulate matter (PM_2.5_) have not been characterized. This study addresses this knowledge gap by introducing a new analytical method to advance the molecular characterization of oxidized D_5_ and its detection in ambient aerosol. The newly developed reversed phase liquid chromatography method, in conjunction with high-resolution mass spectrometry, separates and detects D_5_ oxidation products, enabling new insights into their molecular and isomeric composition. Application of this method to laboratory-generated SOA and urban PM_2.5_ in New York City expands the number of D_5_ oxidation products observed in ambient aerosol and informs a list of molecular candidates to track D_5_-derived SOA in the atmosphere. An oxidation series was observed in which one or more methyl groups in D_5_ (C_10_H_30_O_5_Si_5_) is replaced by a hydroxyl group, which indicates the presence of multistep oxidation products in ambient PM_2.5_. Because of their specificity to PCPs and demonstrated detectability in ambient PM_2.5_, several oxidation products are proposed as molecular tracers for D_5_-derived SOA and may prove useful in assessing the impact of PCPs-derived SOA in the atmosphere.

## Abstract 2

Using a novel analytical method, D_5_ oxidation products were characterized in ambient and laboratory-generated aerosol to develop recommendations for molecular tracers for D_5_-derived SOA.

## Introduction

Volatile methyl siloxanes (VMS) have a silicon-oxygen backbone and methyl substituents on the silicon atoms. This class of compounds, including cyclic and linear methylsiloxanes, are extensively used in personal care products (PCPs) because of their high thermal stability, low surface tension, low viscosity, and high compressibility.^1−4^ Approximately 2.1 million tons of silicone-containing goods were produced in 2013 of which 17% (357000 tons) were personal care and consumer products.^3^ The VMS in PCPs includes hexamethylcyclotrisiloxane (D_3_), octamethylcyclotetrasiloxane (D_4_), decamethylcyclopentasiloxane (D_5_), dodecamethylcyclohexasiloxane (D_6_), hexamethyldisiloxane (L_2_), octamethyltrisiloxane (L_3_), decamethyltetrasiloxane (L_4_), and dodecamethylpentasiloxane (L_5_).^5,6^ D_5_ has been reported as the predominant VMS in personal care products and in the environment.^2,5,7−11^ Prior studies have revealed that over 90% of D_5_ utilized in PCPs is eventually released into the atmosphere.^12,13^ Densely populated urban centers exhibit the highest concentrations of atmospheric VMS, with the lowest concentrations reported in rural and arctic regions.^7,14^

In the atmosphere, D_5_ is oxidized to form oxidized VMS (oVMS) by reacting primarily with OH radicals and to a lesser extent chlorine radicals.^15−17^ In contrast, the loss of D_5_ by reaction with O_3_ and NO_3–_ is predicted to be negligible.^16,17^ The estimated lifetime of D_5_ ranges between 4 to 6 days when exposed to a 24 h average OH radical concentration of 1.2 × 10^6^ molec cm^–3^.^17,18^ Due to the incorporation of oxygen, oVMS products have higher water solubility and lower vapor pressures than their VMS precursors and can partition to the aerosol phase forming secondary organic aerosol (SOA).^1,14,19−22^ oVMS thus have potential to contribute to PCP-derived SOA in urban areas.^23,24^ Although the mass of D_5_-derived SOA on its own is expected to be small relative to the other SOA precursors in PCPs,^25^ products of D_5_ may be useful in tracking SOA derived from PCPs in the atmosphere.

Aerosol generated from VMS oxidation has been characterized in prior studies. 1-Hydroxynonamethylcyclopentasiloxane (D_4_TOH) has been identified as the major D_5_-derived oVMS product.^19^ The gas-particle partitioning of D_4_TOH may depend on aerosol composition and varies with aerosol concentration, humidity, and temperature.^19,26^ Hundreds of additional oVMS products were observed in aerosols collected from D_5_ oxidation in laboratory oxidation experiments.^20,27−29^ These products included ring-opened products, monomers, and dimers. Oxidation introduced functional groups to the VMS, including silanol, silyl alcohol, carboxylic acid, formate esters, and carbonyls. Additionally, dimers connected by O, CH_2_, or CH_2_CH_2_ were found in laboratory studies, either with or without the aforementioned functionalization.^20,27^

While there is extensive research on ambient VMS in the gas phase, measurements of VMS-derived SOA in ambient air have been limited. D_4_TOH was recently observed in ambient fine particulate matter (PM_2.5_) collected in urban areas of Atlanta, Georgia, and Houston, Texas, suggesting its potential as a molecular marker of D_5_-derived SOA in the atmosphere.^30^ Measurements of oVMS in ambient aerosol have been limited by their expectedly low concentrations, lack of authentic standards for identification and quantification, and the capabilities of analytical methodology. While high-resolution mass spectrometry coupled to direct sample infusion with electrospray ionization (ESI) and aerosol mass spectrometry have proven useful in identifying Si-containing molecular formulas of oVMS with high accuracy,^20,27−29^ these techniques are unable to resolve oVMS isomers. Additionally, these techniques measure the components of aerosol samples simultaneously, which may hinder the detection of components at low concentrations, particularly in complex environmental matrices. To date, the quantitative impact of D_5_-derived SOA remains uncharacterized in ambient aerosol and reported measurements of oVMS from D_5_ have been limited to a single product: D_4_TOH.

To advance the analysis of oVMS in ambient aerosol, a novel ultraperformance liquid chromatography–tandem mass spectrometry (UPLC-MS/MS) method was developed. Prior to MS detection, the separation of oVMS using UPLC enabled the resolution and characterization of individual isomers, including determining the placement of siloxanol groups in major isomers. The method developed was applied to characterize major oVMS products in laboratory-generated SOA and ambient PM_2.5_ and to generate a list of molecular candidates to track D_5_-derived SOA in the ambient aerosol in future studies.

## Materials and Methods

### Chemicals and Reagents

Six commercially available organosilicon standards were used as oVMS surrogates for method development: trimethylsilanol (97.5%, Sigma-Aldrich), 1-trimethylsilylmethanol (95%, Thermo Scientific), tris(*tert*-pentoxy)silanol (99.99%, Sigma-Aldrich), tris(*tert*-butoxy)silanol (99.999%, Sigma-Aldrich), dimethylphenylsilanol (95%, Sigma-Aldrich), and *t*-butyldimethylsilanol (99%, Sigma-Aldrich). Acetonitrile was purchased from Fisher Scientific (Optima) and ultrapure water (18.2 MW resistivity) was generated by a water purification system on site (Thermo, Barnsted EasyPure-II). Ammonia (20-22% as NH_3_, Fisher Scientific) and ammonium bicarbonate (99.0%, Sigma-Aldrich) were used to prepare the buffer added to mobile phases to improve ionization.

### Instrumental Analysis Method

An ultraperformance liquid chromatograph (UPLC) interfaced to a Q-Exactive Ultra High Mass Range Hybrid Quadrupole-Orbitrap mass spectrometer (Thermo Scientific) was utilized in negative mode with a heated electrospray ionization source. The following were the optimized ionization conditions for surrogate standards with an optimal buffer system of 10 mM ammonium bicarbonate and ammonium hydroxide at pH 10 and a solvent ratio of acetonitrile to water 50/50 by volume: spray voltage 2.5 kV, capillary temperature 256 °C, vaporizer temperature 413 °C, S-lens frequency 60 Hz, sheath gas flow rate 48 arbitrary units (au), auxiliary gas flow rate 11 au, and sweep gas flow rate 0. For the analysis, two acquisition modes, full scan (FS) and data-dependent MS^2^ (dd-MS^2^) were used. Resolving power of 70000, automatic gain control of (AGC) 1 × 10^6^, and maximum injection time of 200 ms were the full scan optimized settings. The mass range was 64–400 Da for method development utilizing standards and 100–1200 Da for running samples. Resolving power of 17500, AGC of 1 × 10^5^, maximum injection time of 50 ms, and normalized collision energy (NCE) of 35 eV were the optimized dd-MS^2^ settings. A detailed explanation of the optimization procedure that was used to achieve the ionization conditions is provided in the Supporting Information (Figures S1–S3).

Separation was achieved with a reversed-phase BEH C-18 column (Premier ACQUITY UPLC Waters) with dimensions of 100 mm in length, 2.1 mm in diameter, and 1.7 μm in particle size. Ultrapure water with 10 mM ammonium bicarbonate and ammonium hydroxide (pH 10) made up mobile phase A, while 85% acetonitrile and 15% ultra-pure water with 10 mM ammonium bicarbonate and ammonium hydroxide (pH 10) made up mobile phase B. When the amount of water in mobile phase B was less than 15%, the buffer formed a white precipitate. Therefore, mobile phase B was not realistically achievable with a lower water content. The optimized gradient flow is as follows: The gradient’s initial value was 45% B. The B% was maintained at 45% for 0.5 min, increased to 100% at 10 min, and maintained at 100% for 22 minutes. At 22.1 min, the percentage of B returned to 45% and was maintained until 25 min had passed to re-equilibrate the column for the following run. The mobile phase flow rate was 300 μL/min.

### Generation of D_5_ SOA in an Oxidation Flow Reactor

This study compared the chemical composition of SOA generated from D_5_ oxidation in the oxidation flow reactor to SOA collected in New York City (NYC), NY. Aerosols were generated from D_5_ oxidation in a 13 L oxidation flow reactor (OFR, Aerodyne Research, U.S.A.) operated in the OFR185 mode.^31−33^ Aerosol generation was similar to that employed by Janechek et al.^21^ with additional details provided elsewhere.^21,34^ Clean, dry air at 4.5 L min^–1^ (mean residence time of 177 s) was humidified to a relative humidity of 25 ± 2%. Two injection methods were employed: For the first sample collected, liquid D_5_ (97%, Sigma-Aldrich, U.S.A.) was allowed to diffuse from a heated perfluoroalkoxy alkane (PFA) tubing leg into the main flow entering the OFR. Heating was from immersion in a 70° C water bath. In the second sample, D_5_ was introduced at 0.46 ppmv using a 25 μL syringe containing a 20 vol% solution of D_5_ in acetonitrile (99.8%, Sigma-Aldrich, U.S.A.). Experiments were conducted at 28 ± 2 °C, with UV lamps operating at 80% power. No seed aerosols were present during the experiments. Samples were collected onto prebaked (550 °C for 18 h) quartz fiber filters (QFF, 47 mm, PALL Life Sciences). Following collection, QFFs were stored in aluminum foil (prebaked at 550 °C for 5.5 h) lined petri dishes at −20 °C. An OH exposure of 6.8 × 10^12^ molec s cm^–3^ was estimated using simulation of radical chemistry in the OFR.^35,36^ Aerosol concentrations at the point of filter insertion were measured by SMPS and determined to be 218 μg m^-3^ (number concentration was 1.1 × 10^6^ particles cm^–3^, with a predominant particle concentration mode at 40 nm) and 3400 μg m^–3^ (number concentration was 1.5 × 10^6^ particles cm^-3^ with a particle concentration mode at 70 nm), respectively, for the two samples. Sampling conditions were durations were 20 h at 2.5 L min^–1^ and 4.17 h at 1.5 L min^–1^, respectively. Accordingly, estimated loadings on the filters were 0.65 and 1.27 mg, respectively.

### PM_2.5_ Sample Collection

Ambient PM_2.5_ samples were collected on the rooftop of the City University of New York, Advanced Science Research Center, located in Northern Manhattan, New York City (40°48′55.5″N, 73°57′01.5″W), from July 7 to August 3, 2022. This densely populated urban site is surrounded by residential buildings, community buildings (universities and schools), and streets. Samples were collected using two medium-volume air samplers (3000B, URG Corporation), equipped with a cyclone that collects particles with aerodynamic diameter less than 2.5 μm, operating at a flow rate of 90 L min^–1^. The inlets of the samplers were placed approximately 88 m above the mean sea level. During July 7 to July 23, 2022, both the samplers collected PM_2.5_ on prebaked (550 °C for 18 h) QFF (90 mm, PALL Life Sciences). During July 24 to August 3, 2022, samplers collected PM_2.5_ with a two-stage filter holder. The first stage of one sampler collected PM_2.5_ on prebaked (550 °C for 18 h) QFF (90 mm, PALL Life Sciences) while the second stage was equipped with prebaked (550 °C for 18 h) QFF (90 mm, PALL Life Sciences) to collect gas phase oVMS adsorbed to QFF during sampling. The other sampler collected PM_2.5_ with a two-stage filter holder. The first stage collected particulate matter on QFF (90 mm, PALL Life Sciences) while the second stage, used to account for gas phase oVMS adsorption during sampling, utilized precleaned polyurethane foam filters (PUF; URG-2000-30-52PC). Pre- and postsampling flow rates were measured with a calibrated rotameter. PM_2.5_ samples were collected twice daily from 08:00 to 20:00 EDT (daytime) and from 20:00 to 08:00 EDT (nighttime) the following day. Only the first QFF sample with an organic carbon loading of 127.82 μg, measured by the thermal optical analysis,^37^ is analyzed for the purpose of characterization in this study. The sample was collected overnight under clear conditions, with no precipitation. The average wind speed recorded was 4.8 m s^–1^, corresponding to nonstagnant conditions. The best efforts were taken by the staff on the site to refrain from using personal care products during the sampling period and to place the samplers away from any building ventilation. Field blanks were collected every five samples, following the same sampling procedure without passing air through the sampler. Following collection, QFFs were stored in aluminum foil (prebaked at 550 °C for 5.5 h) lined Petri dishes sealed with Teflon tape and stored at −20 °C.

### Sample Preparation and Method Performance

The filter extraction method described by Hettiyadura et al.^38^ was modified to extract oVMS products. The modified procedure is as follows: Samples were extracted twice sequentially (30 min each) with 10.00 mL of acetonitrile and ultrapure water (95:5, by volume) by ultrasonication (60 sonics min^-1^). Extracts were then filtered through polypropylene membrane syringe filters (0.45 μm followed by 0.20 μm pore size, Puradisc, Whatman). The combined filtrate of extraction was reduced to 500 μL under ultrahigh purity nitrogen gas stream (≤5 psi) at 50 °C using an evaporating system (Turbovap LV, Caliper Life Sciences). The extracts were concentrated nearly to dryness at 50 °C using a microscale nitrogen evaporating system (Reacti-Therm III TS 18824 and Reacti-Vap I 18825, Thermo Scientific) after transferring them into 1.5 mL LC vials. Then the extracts were reconstituted with acetonitrile and ultra-pure water (95:5 by volume) to a final volume of 100 μL.

To evaluate the spike recovery, three method blanks and eight spikes were extracted and analyzed. Equation 1 was used to calculate the percentage of spike recovery after subtracting the average blank value. The linear range of quantitation for each standard was determined using a series of standard solutions at 1, 5, 10, 50, 100, 250, and 500 μg L^–1^. The reproducibility of the linear range was determined by repeated analyses of the calibration curves.
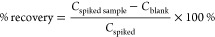
1

### UPLC-MS/MS Data Collection and Analysis

Data were acquired using Xcalibur 4.2 (Thermo Scientific) software. Quantification was performed using the software TraceFinder v4.0 (Thermo Scientific). The detection of at least two ions, one from an MS^1^ scan and the other from a dd-MS^2^ scan, was used as identification criteria for quantification.

Qualitative analysis was performed using Compound Discoverer 3.3.0 (Thermo Scientific) to identify the most abundant ions (Figure S8). First, background peaks in the solvent, method blank, and field blank were removed from consideration. Then, *m*/*z* observed for 103 compounds in OFR experiments in the negative mode by Wu and Johnston^20^ were used to create an inclusion list of oVMS by Compound Discoverer 3.3.0. Reasonable formulas were assigned based on the following criteria: (a) elemental composition falls in the range of C_0-30_H_0-90_O_0-30_Si_0-20_N_0-10_, (b) theoretical *m*/*z* values fall within the ±5 ppm of the observed *m*/*z* value, and (c) observed isotope distributions agreed within 50% with the theoretical isotope distributions for A+1, and A+2 peaks. The most abundant isotopes of Si, including ^28^Si at 92.2%, ^29^Si at 4.7%, and ^30^Si at 3.1%, show a unique isotopic pattern in the mass spectrum for oVMS.

## Results and Discussion

### Method Development

Due to the lack of commercially available authentic standards of oVMS, trimethylsilanol, 1-trimethylsilylmethanol, tris(*tert*-pentoxy)silanol, tris(*tert*-butoxy)silanol, dimethylphenylsilanol, and *t*-butyldimethylsilanol were used as surrogate standards for method development. These compounds were selected because they contain silanol or silylmethanol functional groups expected in oVMS and covered a range of molecular weights (90.20 g mol^–1^ to 306.51 g mol^–1^). Notably, the commercially available surrogate standards lack the Si–O–Si backbone and the O–Si–C sequence, which is expected in oxidized oVMS compounds.

The p*K*_a_ values of the standards with the silanol functional group range from 11 to 15.37, and the p*K*_a_ of the silylmethanol functional group is 15.91. These data indicate that these molecules are very weak acids and do not readily deprotonate. Therefore, a buffer was introduced to aid in the ionization process and facilitate the reproducibility and stability of the ion signal.^39^

Throughout the optimization experiments, 1-trimethylsilylmethanol had the lowest intensity and was often not detected, likely due to its higher p*K*_a_ and lower extent of ionization. Thus, the optimized MS conditions were found to be insufficient to detect silyl methanol functional groups and were instead selective toward silanol functional groups. Acetonitrile was chosen as the organic solvent after considering the standards’ solubility and stability, as well as the solvent’s volatility.

The Premier BEH-C18 column, which retains a wide variety of analytes primarily through strong hydrophobic interactions, was used for separation. Due to the intrinsic chemical stability of ethylene-bridged hybrid particles, this column is suitable for use over a wide pH range from 1 to 12, including the pH of the buffer used in mobile phases (pH 10). Under the optimized UPLC conditions that provided the best resolution of analytes, the aqueous portion of the mobile phase decreased from approximately 62% to 15% and retained the standards within 3 to 12.2 min on the column (Table 1 and Figure S4).

**Table 1:** MS Parameters, LC Retention Time, Linearity, and Coefficient of Determination (*R*^2^)^a^

compound	deprotonated molecule and *m*/*z*		product ions and *m*/*z*		NCE (eV)	retention time (min)	linear range	*R*^2^
tris(*tert*-butoxy)-silanol	C_12_H_27_O_4_Si^–^	263	C_8_H_19_O_4_Si^–^	207	35	8.9	1–500	0.9999
			C_4_H_11_O_4_Si^–^	151				
			H_3_O_4_Si^–^	95				
			HO_3_Si^–^	77				
tris(*tert*-pentoxy)-silanol	C_15_H_33_O_4_Si^–^	305	C_10_H_23_O_4_Si^–^	235	35	12.2	1–500	0.9993
			C_5_H_13_O_4_Si^–^	165				
			H_3_O_4_Si^–^	95				
			HO_3_Si^–^	77				
dimethylphenyl-silanol	C_8_H_11_OSi^–^	151	C_2_H_5_OSi^–^	73	50	3.0	5–500	0.9991
*t*-butyldimethyl-silanol	C_6_H_15_OSi^–^	131	C_3_H_7_OSi^–^	87	-	3.8	50–500	0.9996
trimethyl-silanol	C_3_H_9_OSi^–^	89	C_2_H_5_OSi^–^	73	-	-	-	-
1-trimethylsilyl-methanol	C_4_H_11_OSi^–^	103	-	-	-	-	-	-

a “-” marks *m*/*z* that were not detected.

In contrast, a BEH hydrophilic interaction liquid chromatography (HILIC) column was used in the preliminary studies for separation development. All of the surrogate standards coeluted at retention times of less than 0.6 min (Figure S5), indicating that they did not retain on the BEH HILIC column, but rather migrated with the solvent front. Consequently, the reversed phase column was found to have superior retention, and the HILIC column was discontinued from consideration for this application.

Optimized UPLC-MS/MS conditions were applied to the standards to produce calibration curves within an acceptable range of linearity (*R*^2^ ≥ 0.995), as shown in Table 1. The linearity requirement was met for tris (*tert*-butoxy)silanol and tris(*tert*-pentoxy) silanol across a concentration range of 1–500 μg L^–1^, dimethylphenylsilanol from 5–500 μg L^–1^, and *t*-butyldimethylsilanol from 50–500 μg L^–1^. Trimethylsilanol was below the acceptable range of linearity and suggests a limitation of this method in detecting silanols with low mass-to-charge ratios.

The spike recovery was determined for tris(*tert*-butoxy)silanol, tris(*tert*-pentoxy)silanol, dimethylphenylsilanol, and *t*-butyldimethylsilanol by inputting the results of 3 method blanks and 8 spikes into eq 1 and is summarized below (Table 2). Blanks showed consistent results, with no appreciable concentrations observed. The recovery values for tris(*tert*-butoxy)silanol (72–84%), tris(*tert*-pentoxy)silanol (80–92%), and dimethylphenylsilanol (92–104%) were consistent over eight replicates (RSD = ±9). As recoveries were typically below 100%, it is possible that some of the silanol-containing surrogate standards were chemically lost during the extraction. Hydrolysis is most likely to occur in the surrogate standards at the O-C bond, which has a lower bond energy (85.5 kCal/mol) compared to the Si-O bond (110 kCal/mol). Because the observed and expected oVMS compounds do not contain this Si–O–C sequence (and instead contain siloxanol Si–O–Si and Si–OH), hydrolysis is expected to occur less readily. The recovery of *t*-butyldimethylsilanol decreased below 50 mg L^-1^, which corresponds to the lower boundary of its linear quantification range (LOQ).

**Table 2:** Spike Recoveries Across Eight Spiked Samples Extracted

compound	avg recovery (%)	std deviation	range at 95% confidence interval
tris(*tert*-butoxy)silanol	78	8	72–84
tris(*tert*-pentoxy)silanol	86	9	80–92
dimethylphenylsilanol	98	9	92–104
*t*-butyldimethylsilanol	<LOQ	NA	NA

### Qualitative Analysis of Oxidized Volatile Methyl Siloxanes (oVMS)

oVMS derived from D_5_ were characterized in two samples collected from the OFR laboratory experiments and duplicate samples collected from the NYC field site. Table 3 summarizes the most abundant oVMS ions detected, including their calculated *m*/*z* ([M – H^+^]^−^), the calculated exact mass of the neutral molecule ([M]), the assigned molecular formula ([M]), hydrogen to carbon ratio (H/C), ring plus double bond equivalences (RDBE), and molecular changes from the parent molecule of D_5_ observed under the experimental conditions of this study. It also includes the observed retention time(s) on the reversed phase column, observed mass at each retention time, the error in the exact and observed molecular formulas, identification conditions, and the presence (or absence) of the NYC field sample. A confidence level system used mass spectrometry to communicate the confidence of the proposed molecular identity.^40,41^ Confidence levels range from level 1a–“confirmed by reference standard”, level 2a–“probable structure by library spectrum match”, level 2b–“probable structure by diagnostic homologue and fragmentation evidence”, level 3–“tentative candidate by structure, substituent or class”, level 4–“unequivocal molecular formula,” to level 5–“exact masses of interest”.^40,41^ While it has been shown that D_5_ undergoes dimerization or form higher-order oligomers in OFR experiments,^20,28^ this is rare under typical atmospheric conditions. Consequently, potential tracers for D_5_-derived oVMS were limited to those with 5 silicon atoms or less.

**Table 3:** Summary of the Most Abundant oVMS Ions with Less than 5 Si Atoms Identified in the Laboratory OFR Sample of D_5_ Oxidation^a^

calcd *m*/*z* [M – H^+^]^−^	calcd exact mass [M]	assigned formula [M]	H/C ratio	RDBE	composition changes from the parent molecule D_5_	retention time (*t*_R_, min)	observed *m*/*z*	error [ppm]	identification confidence level	detection in NYC field sample
371.0654	372.0732	C_9_H_28_O_6_Si_5_	3.1	1	-(CH_2_)+(O)	10.9	371.0660	1.65	3	Yes
						11.1	371.0660	1.65	2a	Yes
373.0447	374.0525	C_8_H_26_O_7_Si_5_	3.3	1	-2(CH_2_)+2(O)	7.2	373.0454	2.00	3	Yes
						7.5	373.0452	1.47	3	Yes
						7.9	373.0453	1.73	3	Yes
						8.1	373.0453	1.73	2b	Yes
						8.3	373.0453	1.73	3	No
						16.1	373.0452	1.47	3	No
375.0239	376.0317	C_7_H_24_O_8_Si_5_	3.4	1	-3(CH_2_)+3(O)	2.9	375.0246	1.82	4	No
						3.7	375.0247	2.09	4	No
						4.6	375.0247	2.09	4	No
						5.0	375.0245	1.55	3	No
377.0032	378.0110	C_6_H_22_O_9_Si_5_	3.7	1	-4(CH_2_)+4(O)	3.0	377.0044	3.23	3	No
449.0607	450.06852	C_10_H_30_O_10_Si_5_	3	1	+5(O)	10.5	449.0612	1.12	3	Yes
225.0071	226.0149	C_4_H_14_O_5_Si_3_	3.5	1	-6(C)-16(H)-3(Si)	3.6	225.0078	3.23	4	No
						5.3	225.0077	2.79	3	Yes
297.0466	298.0544	C_7_H_22_O_5_Si_4_	3.1	1	-3(C)-8(H)-(Si)	8.6	297.0471	1.69	3	Yes
						17.7	297.0471	1.69	4	No
						18.1	297.0472	2.02	4	Yes
						18.6	297.0472	2.02	4	Yes
299.0259	300.0337	C_6_H_20_O_6_Si_4_	3.3	1	-4(C)-10(H)+(O)-(Si)	5.1	299.0264	1.80	3	Yes
						5.3	299.0264	1.80	3	Yes
						12.8	299.0264	1.80	4	Yes
301.0051	302.0130	C_5_H_18_O_7_Si_4_	3.6	1	-5(C)-12(H)+2(O)-(Si)	1.4	301.0056	1.57	3	No
						2.3	301.0057	1.90	3	No
302.9844	303.9922	C_4_H_16_O_8_Si_4_	4	1	-6(C)-14(H)+3(O)-(Si)	0.8	302.9849	1.68	3	No
						1.2	302.9846	0.69	3	No

a The last column indicates if the oVMS was detected in the New York City field samples.

**Figure 1:**
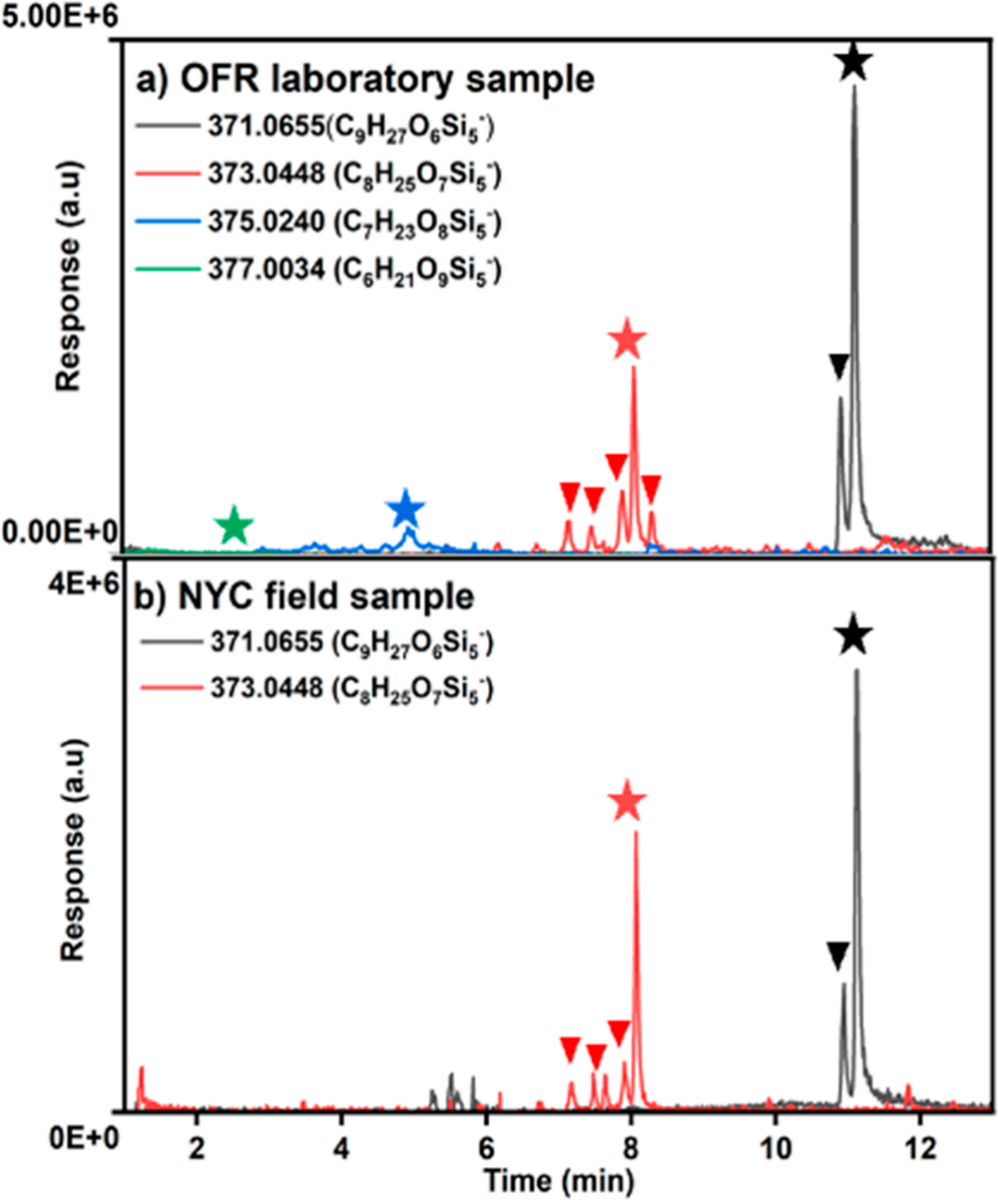
(a) Extracted ion chromatograms of C_9_H_28_O_6_Si_5_ (*m*/*z* 371.0655), C_8_H_26_O_7_Si_5_, (*m*/*z* 373.0448), C_7_H_24_O_8_Si_5_ (*m*/*z* 375.0240), and C_6_H_22_O_9_Si_5_ (*m*/*z* 377.0034) under applied (−)ESI conditions in the OFR laboratory sample and (b) NYC field sample with a tolerance of 5 ppm (most intense peak observed for each *m*/*z* is marked with a star in the OFR sample and the NYC field sample and minor peaks for each *m*/*z* is marked with a triangle).

Four strong ion signals were observed in the OFR samples: C_9_H_28_O_6_Si_5_ (*m*/*z* 371.0655), C_8_H_26_O_7_Si_5_ (*m*/*z* 373.0448), C_7_H_24_O_8_Si_5_ (*m*/*z* 375.0240), and C_6_H_22_O_9_Si_5_ (*m*/*z* 377.0034). Their compositional changes from D_5_ (Table 3) correspond to the replacement of 1, 2, 3, and 4 methyl groups with hydroxyl groups. This oxidation series is consistent with prior OFR experiments^20,27,29^ and indicates the production of multiple generations of D_5_ oxidation products in the OFR experiments. Here, the substitution of one methyl group with a hydroxyl group represents a single oxidation step and generates the first-generation oxidation product, and this pattern continues for subsequent generations. It is noteworthy that each substitution of a methyl group with a hydroxyl group led to a heightened hydrophilic nature of the molecules, consequently resulting in a reduction in their retention time within the C-18 column. This phenomenon is visually depicted in Figure 1a, where distinct colors indicate varying extents of oxidation. The developed UPLC-MS/MS method chromatographically separates of this oxidation series and further reveals the presence of multiple isomers of multi-generation oxidation products. Isomers are indicated by the presence of multiple peaks of the same *m*/*z* in Figure 1a, which are well-resolved in many cases. The product ion spectra acquired for the most prominent isomer within this series are depicted in Figure 3.

C_9_H_28_O_6_Si_5_ (*m*/*z* 371.0655), the first-generation oxidation product, was identified as D_4_TOH in previous work as the major component of D_5_-derived SOA.^19,20,26^ Wu and Johnston^20^ show a possible formation pathway for first- and second-generation oxidation products initiating with a OH abstraction of a hydrogen from D_5_ based on the reaction sequence proposed by Atkinson.^16^ The formation of a multigeneration oxidation product could proceed by either sequential multiple OH abstractions of hydrogen atoms, or by an auto-oxidation process based on internal hydrogen rearrangements.^20^ Depending on the placement of the hydroxyl groups of multigeneration oxidation products, numerous positional isomers are possible (Figure S6). While it is also possible to have isomers with a ring-opened structure and one unit of unsaturation (i.e., Si=O), these are less likely because this would require breaking the relatively strong Si–O bond (110 kCal/mol). Also, Si=O bonds are extremely rare due to the weaker overlap of p orbitals of Si with those of second period elements.^1^

When this series of oVMS oxidation products observed in the OFR is compared with samples from NYC, it becomes evident that only oVMS occurring from the first and second oxidation steps are observed. oVMS undergoing three and four oxidation steps were only detected in the OFR and were not detected in the urban PM_2.5_ sample. The presence of more oxidized products in the OFR is expected to result from greater exposure to OH in the OFR. By considering ambient exposure to OH at a tropospheric concentration of 1.2x10^6^ molec cm^-3^, the calculated approximate age of the OFR sample is 66 days, indicating a much higher degree of aging than is expected for the NYC PM_2.5_ sample.

### Discussion of Identification of Key Isomers of First- And Second-Generation Oxidation Products Using Their Fragmentation Patterns

#### First Generation Oxidation Product of D_5_: C_9_H_28_O_6_Si_5_ (*m*/*z* 371.0655, a.k.a. D_4_TOH)

**Figure 2:**
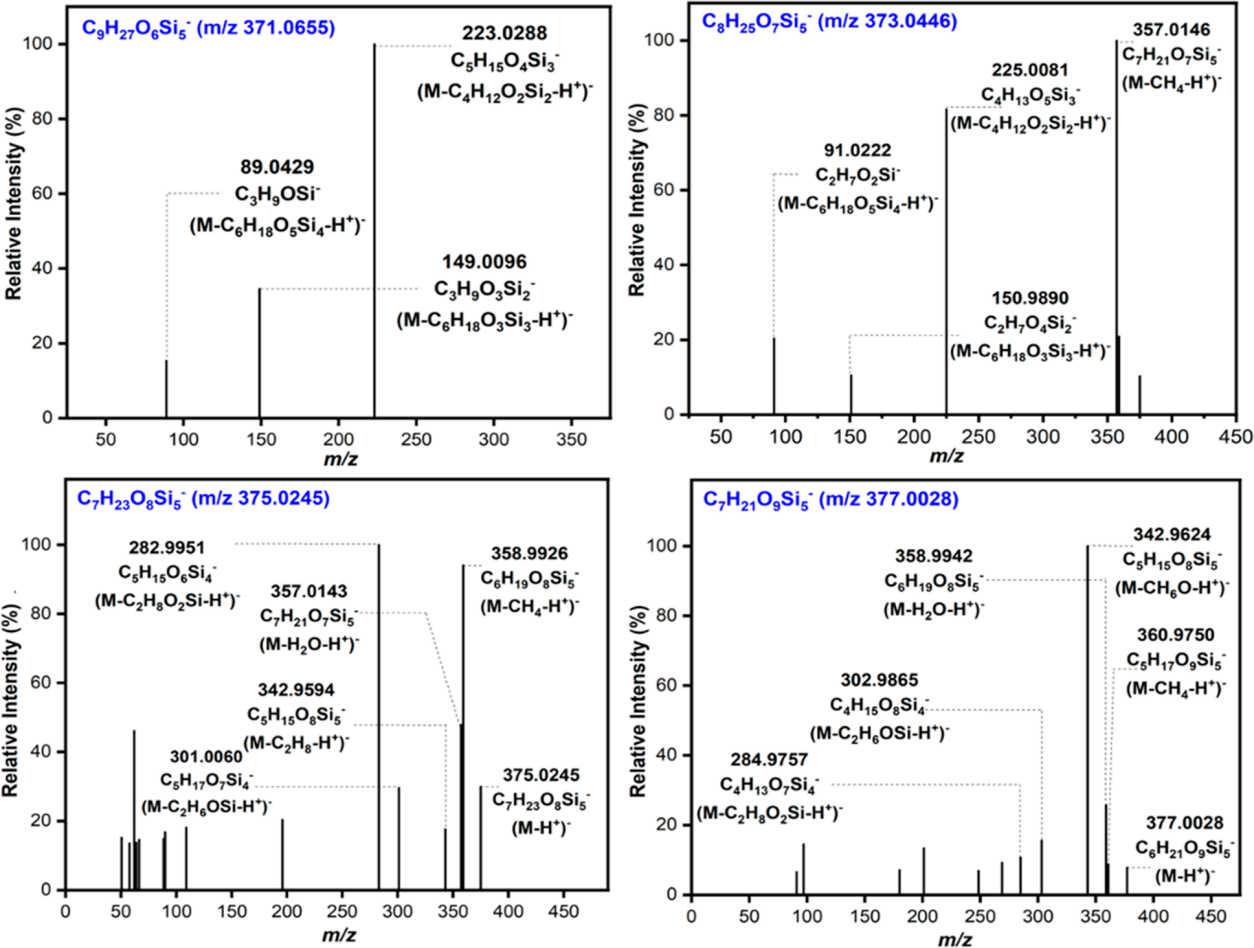
Product ion spectra provided by the (a) C_9_H_28_O_6_Si_5_ (*m*/*z* 371.0655), (b) C_8_H_26_O_7_Si_5_ (*m*/*z* 373.0448), (c) C_7_H_24_O_8_Si_5_ (*m*/*z* 375.0240), and (d) C_6_H_22_O_9_Si_5_ (*m*/*z* 377.0034) under applied (−)ESI conditions for the most intense peak observed in the laboratory OFR sample (marked with stars in Figure 1a).

**Scheme 1:**
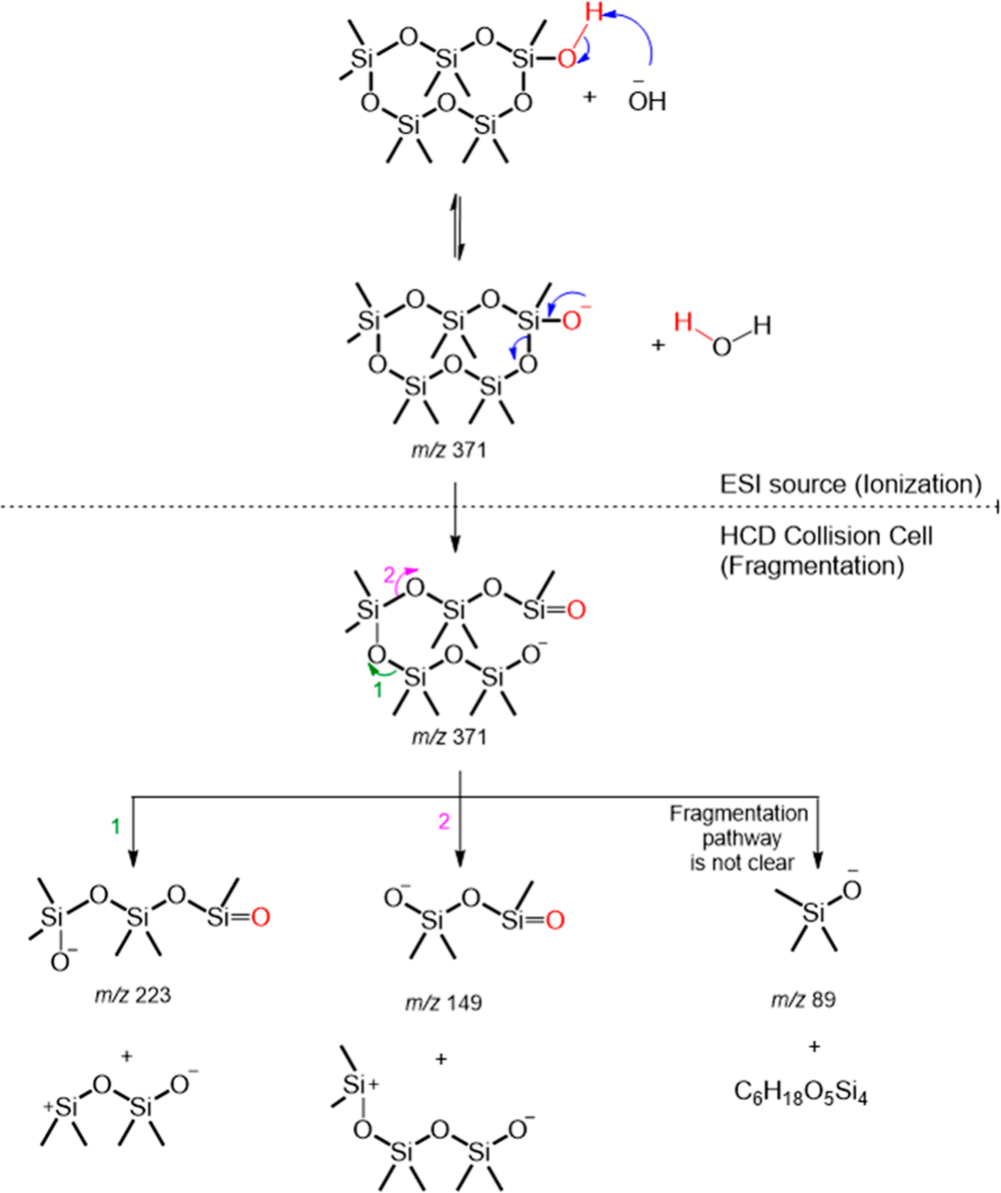
Proposed (−)ESI Fragmentation Pathway for Deprotonated C_9_H_28_O_6_Si_5_ (*m*/*z* 371.0655) This diagram depicts the anticipated deprotonation during ionization, followed by sequential fragmentation within the mass spectrometer collision cell, ultimately yielding the product ion spectra presented in Figure 2a. Accurate mass measurements of the elemental composition of these product ions can be found in Table S1.

There is one positional isomer for C_9_H_28_O_6_Si_5_ with an intact siloxane ring that is formed by replacing a single methyl group of D_5_ with OH (Figure S6). The elemental composition of the product ions was confirmed by accurate mass measurements (Table S1). While commonly referred to as D_4_TOH in the atmospheric chemistry literature, it is more accurately named D_4_D^OH^ because D_4_TOH would describe a silicon subunit containing three half O atoms, but it contains two half oxygen atoms and an OH.^1^ In addition, this nomenclature allows easy differentiation between substitutions.^1,42^ The deprotonated C_9_H_28_O_6_Si_5_ (*m*/*z* 371.0655) showed an intense peak at the retention time of 11.1 min (labeled with a black star in Figure 1a). Its product ion spectrum consists of abundant product ions at *m*/*z* 223 (loss of siloxane subunit C_4_H_12_O_2_Si_2_), *m*/*z* 149 (loss of siloxane subunit C_6_H_18_O_3_Si_3_), and *m*/*z* 89 (loss of siloxane subunit together with a methyl shift) (Figure 2a). The proposed fragmentation pathway of deprotonated C_9_H_28_O_6_Si_5_ is shown in Scheme 1. All these data are consistent with the proposed structure of D_4_D^OH^ (a.k.a. D_4_TOH). The product ion spectrum of C_9_H_28_O_6_Si_5_ (*m*/*z* 371.0655) matched the product ions reported in Wu and Johnston^20^ for the same ion with characteristic peaks at *m*/*z* 223, 149, and 89.

The chromatographic trace for *m*/*z* 371.0655 (deprotonated C_9_H_28_O_6_Si_5_) showed a second, minor peak at a retention time (*t*_R_) of 10.9 min (labeled with a black triangle in Figure 1a). The product ion spectra at *t*_R_ of 10.9 min contains the same fragment ions with approximately the same relative intensities (Figure S7). While its structure is unknown, a plausible structure is a ring-opened product of D_4_D^OH^ with one unit of unsaturation (shown as the ring opened product of *m*/*z* 371 in Scheme 1) because it would be expected to have an equivalent MS^2^ fragmentation pattern. Additionally, a ring-opened product could shift to a chromatographic retention time earlier than that of the ring-retaining siloxane.

When compared to the OFR samples, the PM_2.5_ samples from NYC showed the same chromatographic peaks for *m*/*z* 371.0655 (C_9_H_28_O_6_Si_5_) at *t*_R_ of 11.1 and 10.9 min (Figure 1b) and equivalent to the product ion MS^2^ spectra (Figure 2a). The agreement between the NYC and OFR samples suggests the presence of ring-retaining D_4_D^OH^ and the related product in ambient aerosol. Because of its detectability in this and prior studies, D_4_D^OH^ (a.k.a. D_4_TOH) is a strong candidate to track SOA derived from D_5_.

#### Second Generation Oxidation Product of D_5_: C_8_H_26_O_7_Si_5_ (*m*/*z* 373.0448)

There are three potential positional isomers for neutral C_8_H_26_O_7_Si_5_ maintaining an intact siloxane ring, when considering that two methyl groups of D_5_ are replaced by OH: D_3_-(1,2)-D^OH^_2_ (vicinal siloxanol), D_3_-(1,3)-D^OH^_2_ (free siloxanol), and D_4_D^(OH)2^ (geminal siloxanol, Figure S6).

**Figure 3:**
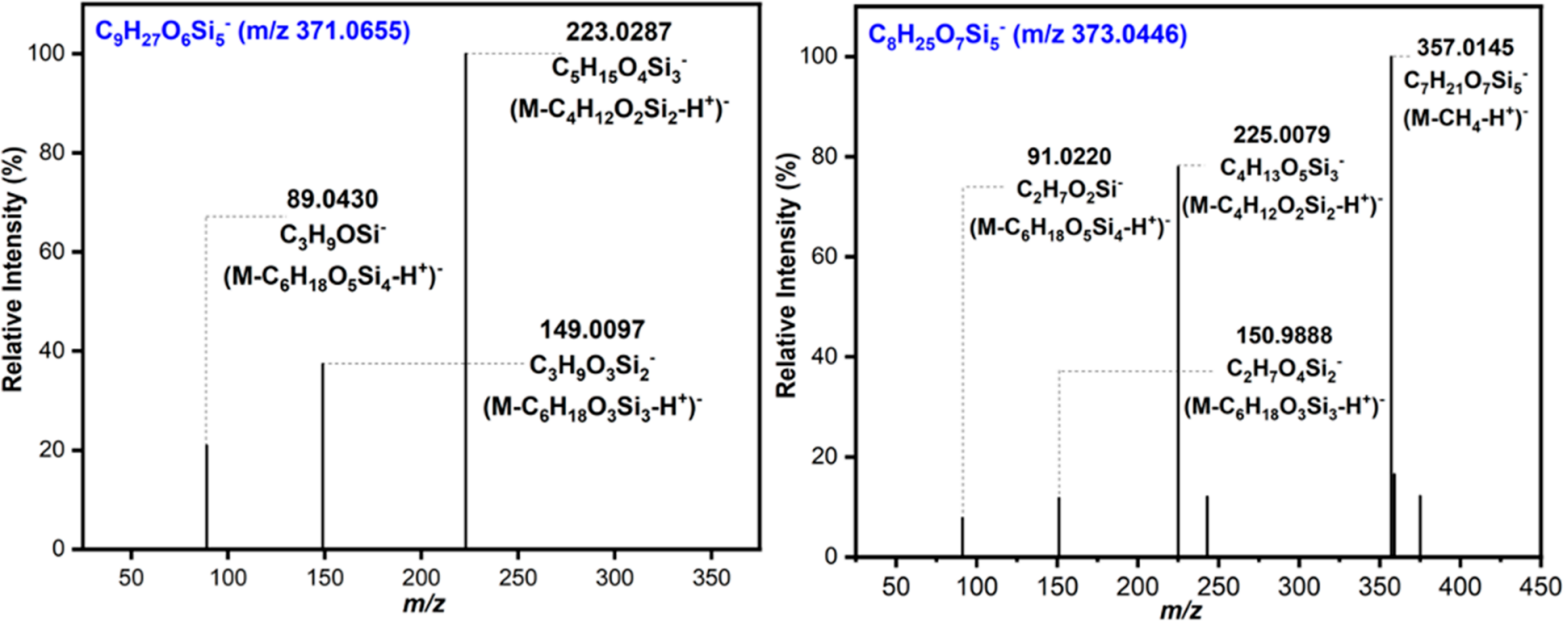
Product ion spectra provided by the (a) C_9_H_28_O_6_Si_5_ (*m*/*z* 371.0655), (b) C_8_H_26_O_7_Si_5_ (*m*/*z* 373.0448), under applied (−)ESI conditions for the most intense peak observed in the NYC field site sample (marked with a star in Figure 1b).

**Scheme 2:**
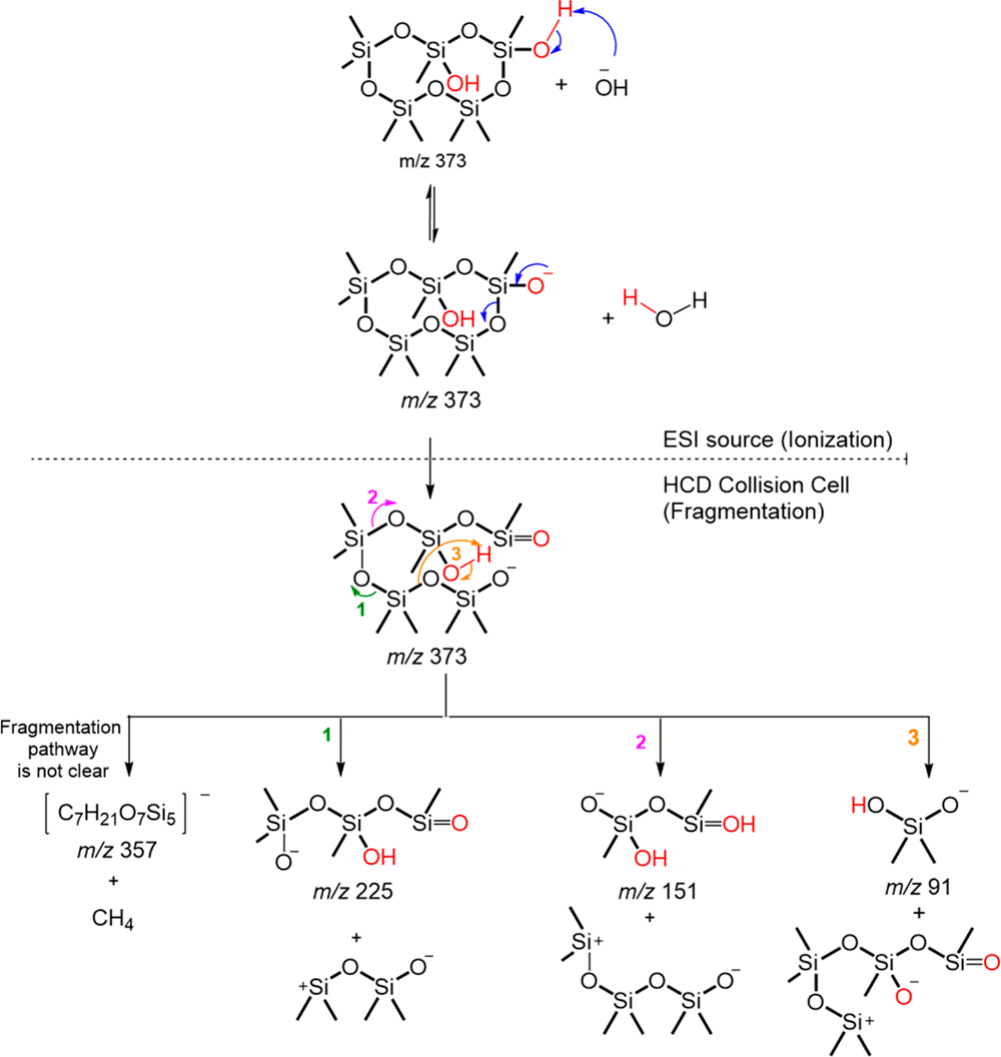
Proposed Fragmentation Pathway of D_3_-(1,2)-D^OH^_2_ This diagram depicts the anticipated deprotonation during ionization, followed by sequential fragmentation within the collision cell, ultimately yielding the product ion spectra presented in Figure 2b. Accurate mass measurements of the elemental composition of these product ions can be found in Table S1.

The deprotonated C_8_H_26_O_7_Si_5_ (*m*/*z* 373.0448) showed an intense peak at the retention time of 8.1 min (labeled with a red star in Figure 1a). The product ion spectrum consists of ions at *m*/*z* 357 (successive neutral loss of CH_4_), *m*/*z* 225 (successive loss of siloxane subunits of C_4_H_12_O_2_Si_2_), *m*/*z* 151 (successive loss of siloxane subunits of C_6_H_18_O_3_Si_3_), and *m*/*z* 91 (successive loss of siloxane subunits of C_6_H_18_O_5_Si_4_) (Figure 2b), with the elemental composition of product ions confirmed by accurate mass measurements (Table S1). The proposed fragmentation pathway of deprotonated C_9_H_28_O_6_Si_5_ is shown in Scheme 2 and supports the proposed structure of D_3_-(1,2)-D^OH^_2_, in which siloxanol groups occur on adjacent Si atoms. This MS^2^ spectrum could not correspond to D_3_-(1,3)-D^OH^_2_, with siloxanols on nonadjacent Si atoms, because the successive loss of C_6_H_18_O_3_Si_3_ can only occur in molecules with three consecutive Si(CH_3_)_2_O- in the siloxane ring. Additionally, this MS^2^ spectrum is not expected to correspond to geminal siloxanol D_4_D^(OH)2^ because of the absence of the neutral loss of H_2_O (*m*/*z* 18) that is expected from geminal siloxanol. The observation and high relative abundance of this peak in the NYC PM_2.5_ sample at a retention time of 8.1 min (Figure 1b) and matching product ion spectrum (Figure 3b) support the identification of D_3_-(1,2)-D^OH^_2_ as the major isomer of the second-generation D_5_ oxidation product.

Four additional chromatographic peaks for *m*/*z* 373.0448 (deprotonated C_8_H_26_O_7_Si_5_) were observed at 7.2, 7.5, 7.9, and 8.3 min in the OFR sample (labeled with red triangles in Figure 1a). Product ion spectra, however, could not be obtained due to their low relative abundance, even with an increased injection volume of 20 μL. Among these, peaks at 7.2, 7.5, and 7.9 min were consistently observed in the NYC sample (Figure 1b). It is expected that these minor peaks correspond to ring-retaining D_3_-(1,3)-D^OH^_2_ and D_4_D^(OH)2^ or the ring-opened positional isomers of these; however, this could not be confirmed.

#### Other Oxidation Products of D_5_

Additional oVMS ions were detected in the laboratory OFR samples and ambient PM_2.5_ from NYC (Table 3). C_10_H_30_O_10_Si_5_ (*m*/*z* 449.0608) was observed in OFR samples and is expected to be an oxidized monomer of D_5_ that could be formed replacing methyl groups with CH_2_OH or CH_2_OOH. Among these, the peroxyl functional group is considered to be unlikely because the CH_2_OOH would likely decompose during sample collection, extraction, and/or analysis because of its relatively weak O–O bond energy of 34 Kcal/mol.^43,44^ C_4_H_14_O_5_Si_3_ (*m*/*z* 225.0072), C_7_H_22_O_5_Si_4_ (*m*/*z* 297.0476), C_6_H_20_O_6_Si_4_ (*m*/*z* 299.0259), C_5_H_18_O_7_Si_4_ (*m*/*z* 301.0049), and C_4_H_16_O_8_Si_4_ (*m*/*z* 302.9844) are oxidized and fragmented products of D_5_, which either retain the cyclic siloxane backbone or undergo ring-opening and maintain one unit of unsaturation. Alternatively, C_7_H_22_O_5_Si_4_ (*m*/*z* 297.0476), C_6_H_20_O_6_Si_4_ (*m*/*z* 299.0259), C_5_H_18_O_7_Si_4_ (*m*/*z* 301.0049), and C_4_H_16_O_8_Si_4_ (*m*/*z* 302.9844) could form from the oxidation of D_4_, forming first through fourth-generation oxidation products, respectively, that are analogous to those observed for D_5_ and discussed above. Detected in ambient PM_2.5_ and OFR-generated SOA, C_8_H_26_O_9_Si_6_ (*m*/*z* 433.0120) and C_10_H_32_O_8_Si_6_ (*m*/*z* 447.0638) contain six Si atoms and could have originated from D_6_ precursor that is an expected contaminant in 97% pure D_5_.^45,46^ or from D_5_ accretion reactions. D_6_ is also an observed contaminant from PCPs in the atmosphere, as mentioned in the introduction, though it is not as prominent as D_5_. Among the other oxidation products in Table 3, C_10_H_30_O_10_Si_5_ (*m*/*z* 449.0608), C_4_H_14_O_5_Si_3_ (*m*/*z* 225.0072), C_7_H_22_O_5_Si_4_ (*m*/*z* 297.0476), C_6_H_20_O_6_Si_4_ (*m*/*z* 299.0259), were detected in the OFR and ambient PM_2.5_ samples. These molecules may also prove to be useful in understanding the mechanisms of VMS oxidation and/or tracking personal-care-product-derived SOA in ambient air.

#### Recommendation for Molecular Tracers of D_5_

**Figure 4:**
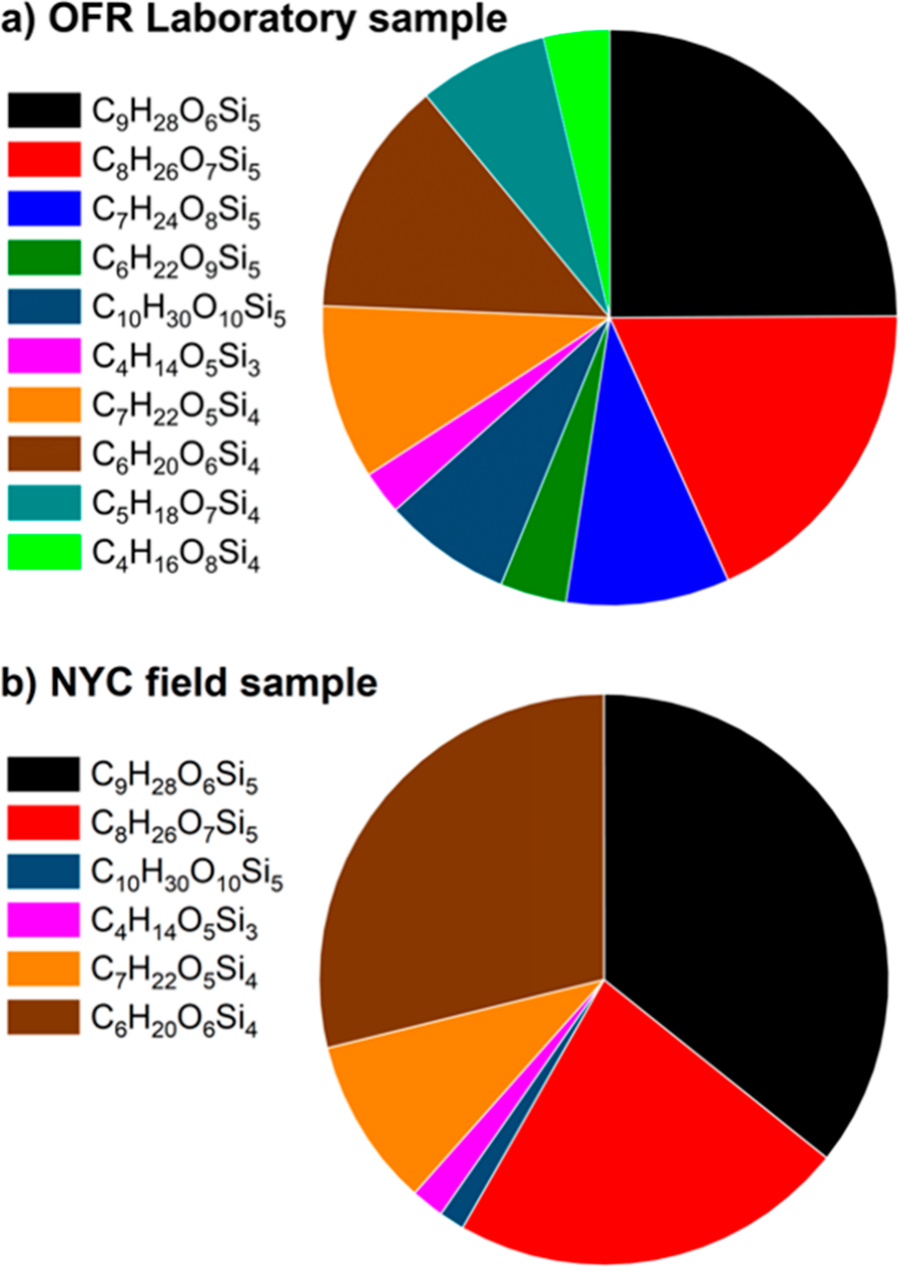
Relative signal abundance from major oVMS signals observed in (a) D_5_-derived SOA generated by the OFR and (b) NYC PM_2.5_. Details of the listed formulas are provided in Table 3.

SOA tracers ideally provide specificity to a precursor gas, are stable in the atmosphere, partition to the particle phase, and are readily and reliably quantified. Among the detected ions in Table 3, D_4_D^OH^ (a.k.a. D_4_TOH) and D_3_-(1,2)-D^OH^_2_, identified as the first and second oxidation products of D_5_, hold the greatest promise as molecular tracers of D_5_-derived SOA. Currently, their molecular structures are most confidently assigned, based on chromatographic, MS, and MS/MS data (Table 3). These molecules have also been identified in prior studies of D_5_-derived SOA, supporting their origin being from this source.^19,20,26,28^ Because these molecules retain the cyclic VMS backbone, they are readily distinguished from linear VMS precursors. In regard to signal abundance, these two ions corresponding to D_4_D^OH^ and D_3_-(1,2)-D^OH^_2_ are among the greatest oVMS signals detected from D_5_ in the OFR laboratory sample and in NYC PM_2.5_ (Figure 4). Additionally, D_4_D^OH^ (a.k.a. D_4_TOH) has previously been observed in urban aerosol,^30^ further demonstrating detectability in ambient PM_2.5_. The strong MS signals for these molecules will be useful in their quantitative analysis by enabling greater detectability and sensitivity and lower detection limits. For these reasons, D_4_D^OH^ (D_4_TOH) and D_3_-(1,2)-D^OH^_2_ molecules should be prioritized for standard development and quantification. Other oVMS listed in Table 3 should undergo further characterization to increase confidence in their structures prior to standard development.

## Conclusion

To advance the ability to detect oVMS in ambient aerosols, a novel and sensitive UPLC-MS/MS method was developed. The use of optimized electrospray ionization source conditions and the selected solvent system, buffer composition, concentration, and pH yielded high sensitivity and reproducibility for the silanol functional group. The developed method was able to detect surrogate standards with silanol functional groups with good linearity (*R*^2^ > 0.999) and linear ranges of 1–500 μg L^-1^ for silanols in the molecular weight range of D_5_ (i.e., *m*/*z* 263 and 305). The method was validated for semi-quantification by eight spike recovery experiments of surrogate standards, with recoveries showing consistency (RSD = ±9%) for tris(*tert*-butoxy) silanol (72–84%), tris(*tert*-pentoxy)silanol (80–92%), and dimethylphenylsilanol (92–104%). The precision of the extraction and analysis method (RSD ±9) will support precise measurements of oVMS in future quantitative analysis of oVMS in aerosol. Notably, the limitations of this method include insensitivity in detecting the silyl methanol functional group attached to silicon and low-molecular weight silanols (< 100 amu). The utility of the developed UPLC-MS method is demonstrated herein for characterization of oxidation products from the OH oxidation of D_5_, and is expected to be well suited to examine chemically similar precursors (i.e. D_4_, D_6_) and other oxidants.

The integration of UPLC with electrospray ionization MS has advanced our understanding of oVMS derived from D_5_ in the atmosphere. Notably, this study marks the first measurement of oVMS from D_5_ beyond D_4_TOH in an ambient aerosol. In combination with high resolution MS, UPLC retention time increases our ability to determine which oVMS (and which oVMS isomers) that are generated in the laboratory OFR are also detected in ambient aerosol. Additionally, this method has generated new insights into the number and structure of major oVMS isomers. Among the observed oVMS, C_9_H_28_O_6_Si_5_ (*m*/*z* 371.0655), and C_8_H_26_O_7_Si_5_ (*m*/*z* 373.0448) are identified as D_4_D^OH^ (D_4_TOH) and D_3_-(1,2)-D^OH^_2_, respectively, and form an oxidation series via the replacement of one or more methyl groups in D_5_ by a hydroxyl group. Up to four oxidation steps were observed in the OFR samples, while two oxidation steps were detected in the NYC PM_2.5_ samples, suggesting that multigeneration oxidation products are readily formed under high OH exposures in the OFR, while fewer oxidation steps occur in the ambient environment. Because of their specificity to D_5_, relatively high signal abundance (Figure 4), and demonstrated detectability in ambient PM_2.5_, these compounds should be prioritized for future standard development for the confirmation of compound identity and absolute quantitation. Although D_5_ is not expected to be the main cause of SOA in the urban atmosphere, these tracers may prove to be useful in assessing and constraining the contributions of PCPs to SOA.

## Supporting Information Available

Response of the mixed standard solution at three different buffer compositions and three different solvent ratios (Figure S1) and two different buffer concentrations and buffer pH (Figure S2), source parameter optimization conditions of Q-Exactive mass spectrometer, mobile phase optimization conditions, product ion spectra provided by the standards under applied (−)ESI conditions (Figure S3), extracted chromatograms from a mixed solution containing each of four standards that was detected and was in the accepted range of linearity (*R*^2^ ≥ 0.995) in the C-18 column (Figure S4) and in the HILIC column (Figure S5), possible isomers for C_9_H_28_O_6_Si_5_ (*m*/*z* 371.0655), C_8_H_26_O_7_Si_5_ (*m*/*z* 373.0448), C_7_H_24_O_8_Si_5_ (*m*/*z* 375.0240), and C_6_H_22_O_9_Si_5_ (*m*/*z* 377.0034) (Figure S6), product ion spectra provided by the (a) C_9_H_28_O_6_Si_5_ (*m*/*z* 371.0655) under applied (−)ESI conditions for the peak observed in the OFR sample at *t*_R_ 10.9 min (Figure S7), the elemental composition of product ions observed by the most intense peak of laboratory OFR sample (marked with stars in Figure 1a and Table S1), comprehensive flow diagram outlining the qualitative data analysis process for data acquired through UPLC-MS/MS analysis, covering both laboratory and New York City (NYC) samples (Figure S8) (PDF)
