# Supplementary material for: Advances in the Separation and Detection of Secondary Organic Aerosol Produced by Decamethylcyclopentasiloxane (D5) in Laboratory-Generated and Ambient Aerosol
Source: ACS EST Air. 2024 Mar 27;1(5):365–75. doi: 10.1021/acsestair.3c00073 (PMC11091883; doi:10.1021/acsestair.3c00073)
Supplement: Supplementary file 1 — ea3c00073_si_001.pdf [file ea3c00073_si_001.pdf]

Supporting information for:

Advances in the separation and detection of secondary organic aerosol produced by decamethylcyclopentasiloxane (D_5) in laboratory-generated and ambient aerosol

Jeewani N. Meepage[1], Josie K. Welker[1], Claire M. Meyer[1], Saeideh Mohammadi[2], Charles O. Stanier[2], Elizabeth A. Stone[1,2]

[1]University of Iowa, Department of Chemistry, Iowa City, Iowa 52242 USA

[2]University of Iowa, Department of Chemical and Biochemical Engineering, Iowa City, Iowa 52242 USA

This file includes:

Number of pages 10

Number of figures: 8

Number of tables:1

Supporting information:

Mobile phase optimization

For mobile phase optimization, the response of surrogate standards was assessed under three different solvent ratios of acetonitrile to water (60:40, 50:50, and 40:60), three different buffer compositions (20 mM ammonium acetate, 20 mM ammonium hydroxide with ammonium bicarbonate, and 20 mM ammonium hydroxide), two different concentrations (10 mM and 20 mM), and two pH values (10 and 11).

The effect of the acetonitrile-to-water solvent ratio on the response of surrogate standards at 1000 μg L^{-1} each was examined at 60:40, 50:50, and 40:60. There were no significant differences in intensities (Figure S1) and the 50:50 acetonitrile-to-water ratio was used for subsequent optimization.

Three buffer systems, ammonium acetate (pH 6.67), ammonium hydroxide with ammonium carbonate (8.63), and ammonium hydroxide (9.95), were examined at concentrations of 20 mM in a surrogate standard solution at 1000 μg L^{-1}. Among these, 20 mM ammonium hydroxide resulted in the highest intensities for all six standards compared to the other two buffer systems (Figure S1). It is expected that the highest intensities were achieved for ammonium hydroxide because its pH is the most basic and is closest to the pK_a values of the standards. To increase buffer capacity, ammonium bicarbonate (20 mM) was added. When the pH was increased from pH 10 to pH 11, there was no further increase in intensity (Figure S2), so the lower pH value of 10 was utilized. The buffer concentration was lowered from 20 mM to 10 mM and the intensity of the signals did not significantly decrease (Figure S2), so the 10 mM buffer concentration was utilized. Having a lower concentration of buffer pH is generally preferred in ESI mass spectrometry to improve sensitivity by decreasing the number of ions created from the background compounds.

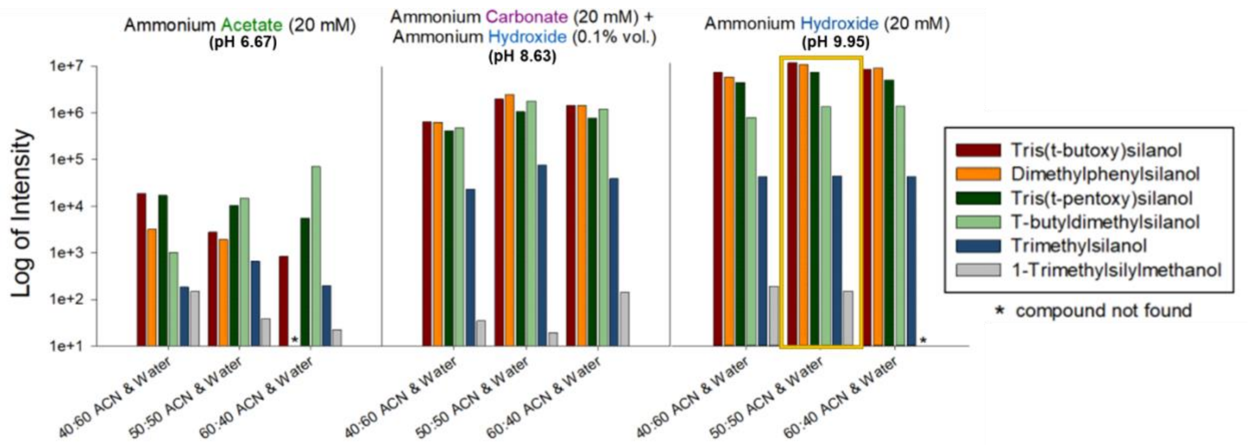

Figure S1: Log of the intensity of the mixed standard solution of 1000 μg L^{-1} at three different buffer compositions and three different solvent ratios

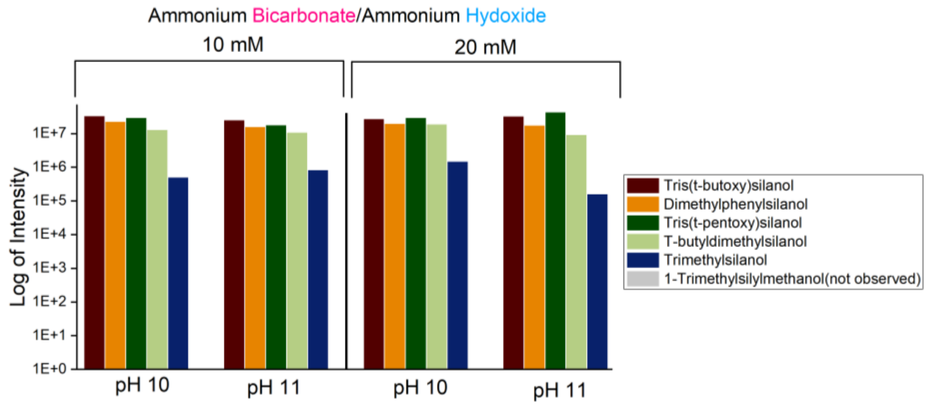

Figure S2: Log of the intensity of the mixed standard solution of 1000 μg L^{-1} at two different buffer concentrations (20 mM and 10 mM) and two pH (11 and 10).

Additional source parameters

A 1000 μg L^{-1} solution of standards was introduced into the mass spectrometer through direct infusion while mobile phase flow was maintained via a T-junction in order to optimize the ESI source parameters that control the spraying of the LC eluent and negative ionization of the molecules. Because those parameters are affected by flow rate and mobile phase composition, a T-connection was necessary to simulate chromatographic conditions. The sheath gas is the inner nitrogen flow that nebulizes the sample flowing from the needle to form fine droplets, whereas the auxiliary gas is the heated outer nitrogen flow that aids the sheath nitrogen in sample desolvation.

The temperature of the heater was kept at 413 °C to achieve optimal sample desolvation. A spray voltage of 2.5 kV in negative mode was used to achieve ionization. The ion transfer capillary is responsible for transferring the ionized species generated by the ESI source to the S-lens while also ensuring that any residual solvent is evaporated. The temperature of the capillary was kept constant at 256 °C.

A 1000 μg L^{-1} standard solution was directly infused to acquire data-dependent MS^2 (dd-MS^2) data (Table 1). In the data-dependent acquisition mode, each precursor with an isolation window of 1 Da was isolated, fragmented, and its product ions are detected. The obtained data were used to assign formulas to deprotonated molecules and product ions. Product ion spectra provided by the above standards under applied (-) ESI conditions are shown in Figure S3. Tris (tert-butoxy)silanol (Figure S3a) fragmented to *m/z* 207, 151, and 95 fragments from the H abstraction followed by the heterolytic cleavage of each O-C bond in Si-O-C bond sequence forming a silanol functional group. The bond energy of Si-O is higher (110 kCal/mol) compared to the bond energy of O-C (85.5 kCal/mol) leading to preferential breaking of the O-C bond during the fragmentation. A similar fragmentation pattern was observed in tris (tert-pentoxy) silanol (Figure S3b).

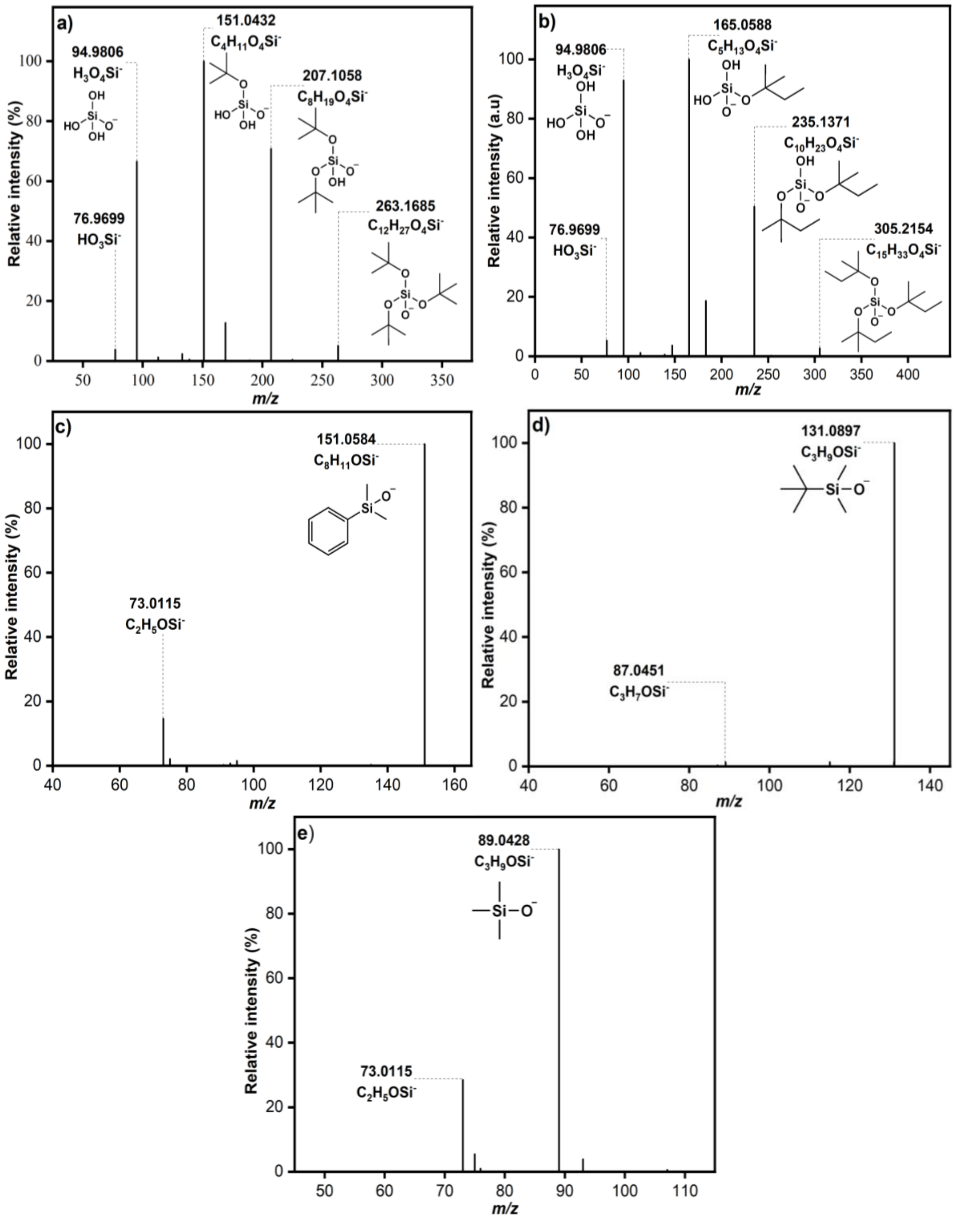

Figure S3: Product ion spectra provided by the standards a) tris (tert-butoxy)silanol, b) tris(tert-pentoxy)silanol, c) dimethylphenylsilanol, d) t-butyldimethylsilanol and e) trimethylsilanol under applied (-)ESI conditions

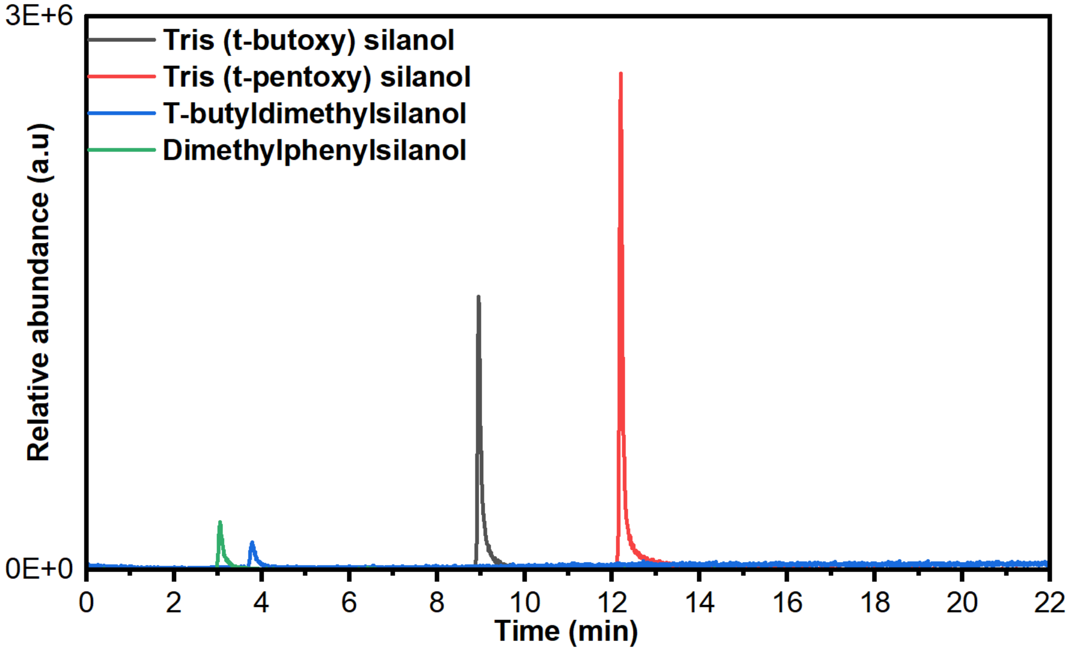

Figure S4: Extracted chromatograms from a mixed solution containing 100 µg L^{-1} of each of four standards that was detected and was in the accepted range of linearity ($R^2 \geq 0.995$) in the C-18 column

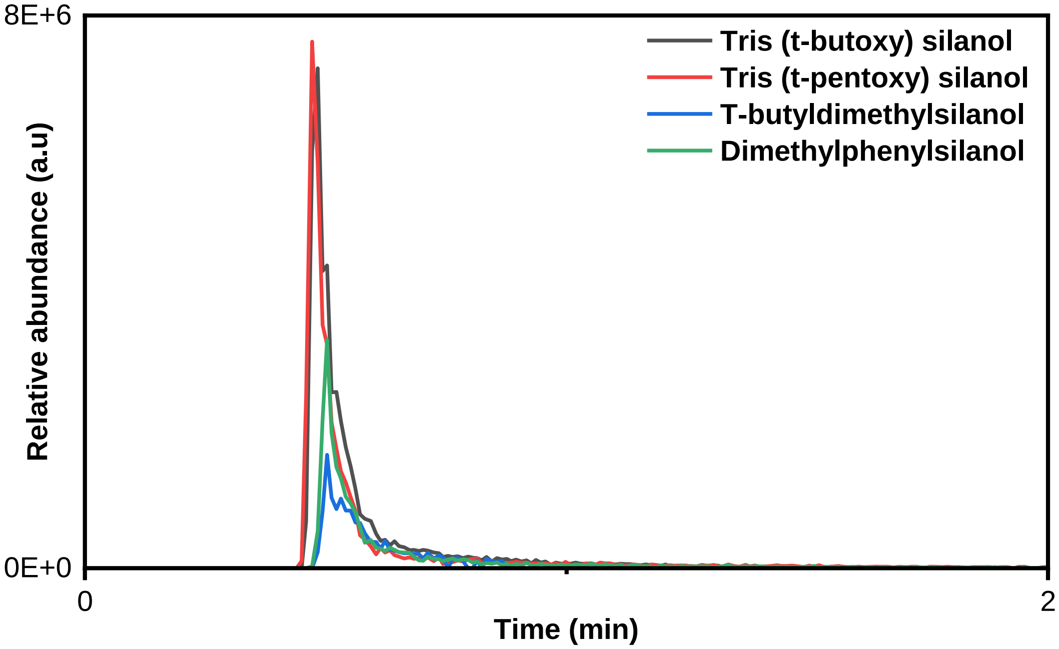

Figure S5: Extracted chromatograms from a mixed solution containing 500 µg L^{-1} of each of four standards that was detected and was in the accepted range of linearity ($R^2 \geq 0.995$) in the HILIC column. Poor retention led to the use of reversed phase liquid chromatography as an alternative approach.

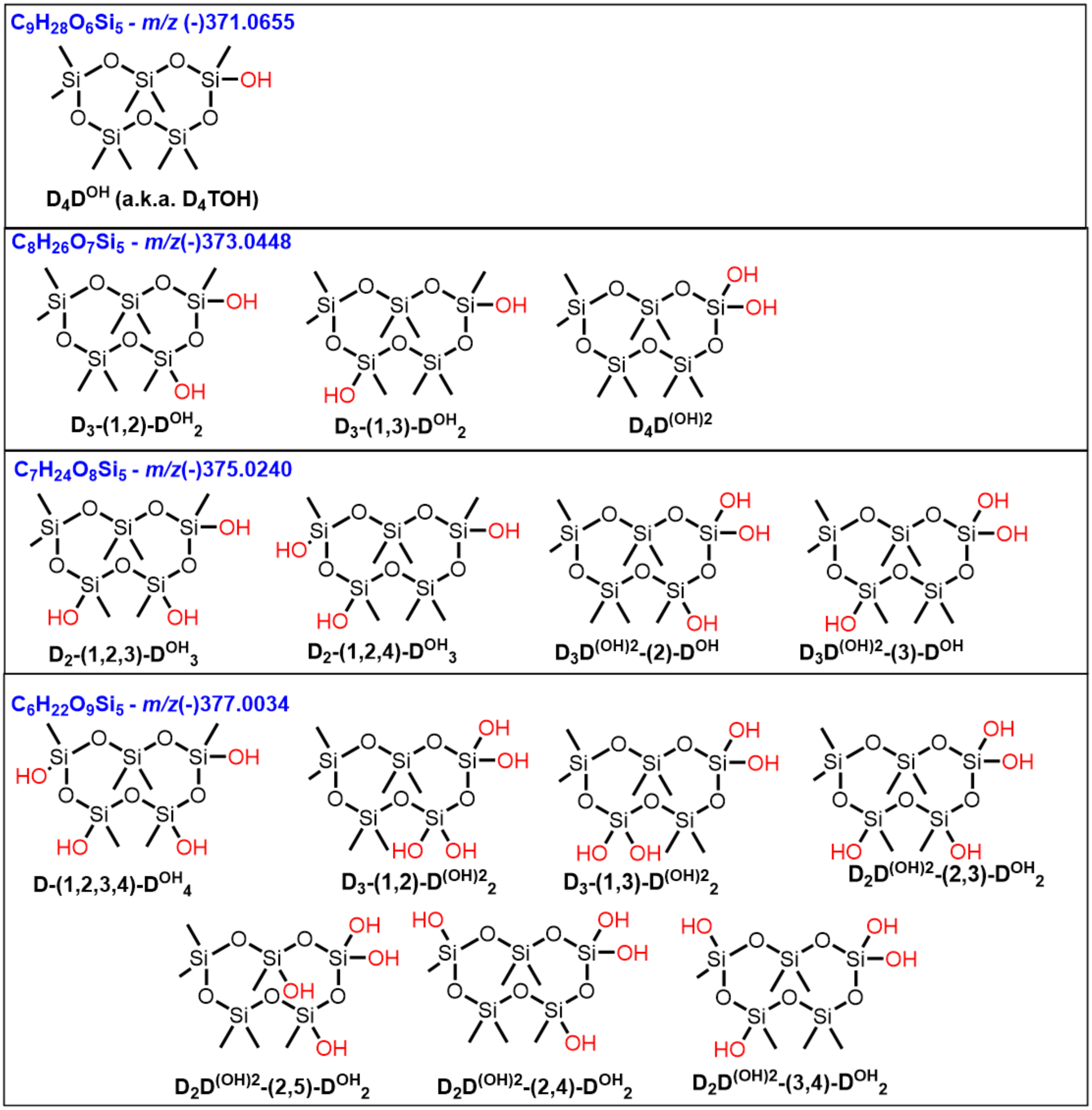

Figure S6: Following the notation for methyl siloxanes in a prior reviews,(Brook 2000, Rucker and Kummerer 2015) each possible isomer is given a notation in which D stands for divalent $Me_2SiO_{2/2}$ (difunctional silicon), and the functional group superscript to the right of the symbol D denotes the group that substitutes the methyl group. The superscripted number indicates that a single silicon would be substituted more than once with the specified functional group (e.g., in the case of a geminal silanol). The subscripted number represents the number of occurrences of the substituted silicon appears, while the preceding number(s) inside parenthesis indicates the position(s) of the substituted silicon.

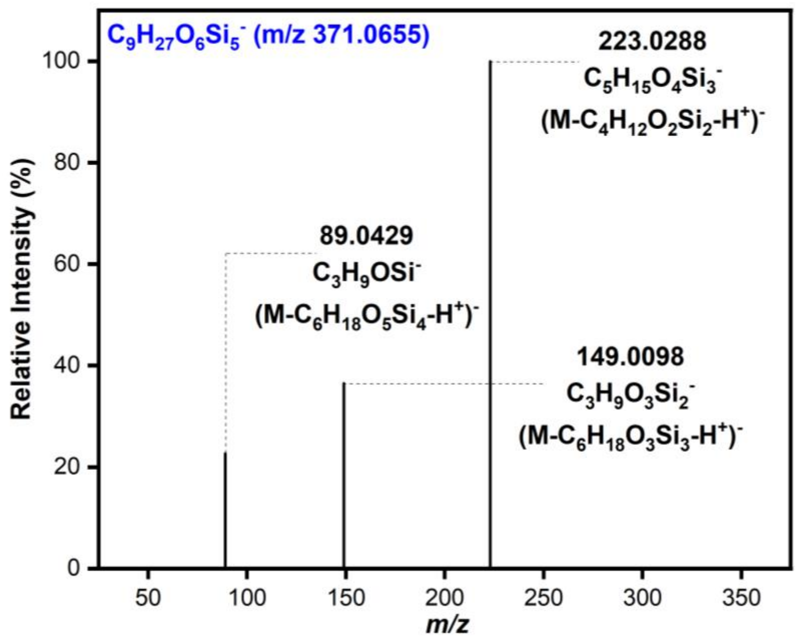

Figure S7: Product ion spectra provided by the a) $C_9H_{28}O_6Si_5$ (*m/z* 371.0655) under applied (-) ESI conditions for the peak observed in the OFR sample at t_R 10.9 min

Table S1: The elemental composition of product ions observed by the most intense peak of laboratory OFR sample (marked with a star in Figure 1a).

**Oxidation product**	**Ion formula (-) ESI**	**Calculated (*m/z*)**	**Observed (*m/z*)**	**Error (ppm)**
D_4D^{OH}	$C_9H_{27}O_5Si_5^-$	371.0654	371.0655	0.30
	$C_5H_{15}O_4Si_3^-$	223.0278	223.0288	4.44
	$C_3H_9O_3Si_2^-$	149.0090	149.0096	3.90
	$C_3H_9OSi^-$	89.0423	89.0429	7.13
D_3-(1,2)-$D^{OH}{}_2$	$C_8H_{25}O_5Si_5^-$	373.0447	373.0446	-0.14
	$C_7H_{21}O_7Si_5^-$	357.0134	357.0146	3.49
	$C_4H_{13}O_5Si_3^-$	225.0071	225.0081	4.56
	$C_2H_7O_4Si_2^-$	150.9883	150.9890	4.75
	$C_2H_7O_2Si^-$	91.0215	91.0222	7.37
$C_7H_{24}O_8Si_5$	$C_7H_{23}O_8Si_5^-$	375.0239	375.0245	1.55
	$C_6H_{19}O_8Si_5^-$	358.9926	358.9926	-0.05
	$C_7H_{21}O_7Si_5^-$	357.0134	357.0143	2.65
	$C_5H_{15}O_8Si_5^-$	342.9613	342.9594	-5.59
	$C_5H_{17}O_7Si_4^-$	301.0051	301.0060	2.90
	$C_5H_{15}O_6Si_4^-$	282.9946	282.9951	1.90
$C_6H_{22}O_9Si_5$	$C_6H_{21}O_9Si_5^-$	377.0032	377.0028	-1.01
	$C_5H_{17}O_9Si_5^-$	360.9719	360.9750	8.64
	$C_6H_{19}O_8Si_5^-$	358.9926	358.9942	4.41
	$C_5H_{15}O_8Si_5^-$	342.9613	342.9624	3.16
	$C_4H_{15}O_8Si_4^-$	302.9844	302.9865	6.96
	$C_4H_{13}O_7Si_4^-$	284.9738	284.9752	4.82

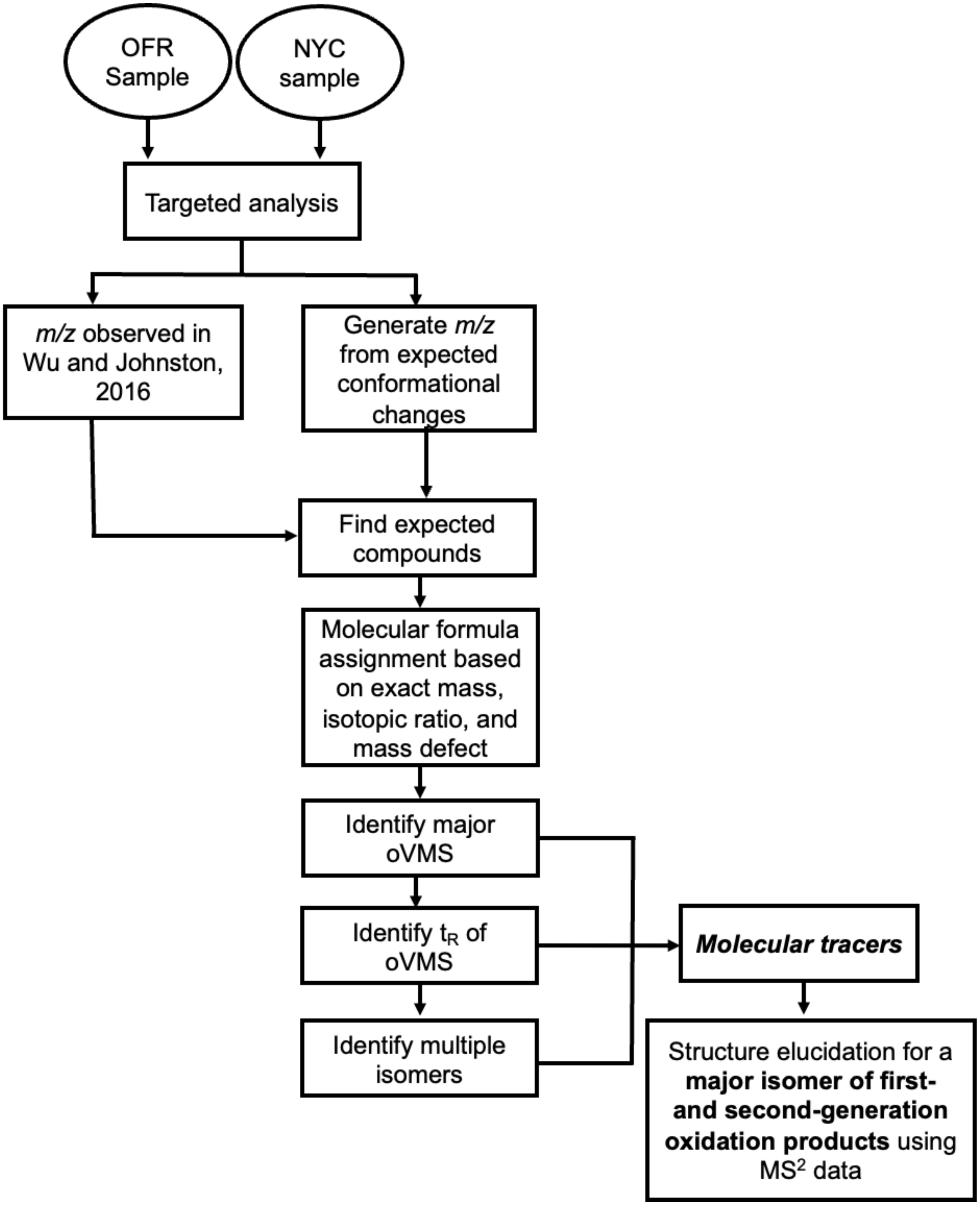

Figure S8: Comprehensive flow diagram outlining the qualitative data analysis process for data acquired through LC-MS analysis, covering both laboratory and New York City (NYC) samples.
